# Options for financing pandemic preparedness

**DOI:** 10.2471/BLT.17.199695

**Published:** 2017-12-01

**Authors:** Patrick L Osewe

**Affiliations:** a Health, Nutrition and Population, World Bank Group, 1818 H Street, NW, Washington DC, 20433, United States of America.

The health, economic and social impacts of disease outbreaks are persistent and manifold. Lessons learnt from the 2013–2016 Ebola virus disease outbreak in West Africa demonstrate how far-reaching the cost of pandemics can be. Beyond the devastating death toll of 11 310,^1^ Guinea, Liberia and Sierra Leone lost 2.2 billion United States dollars (US$) in gross domestic product (GDP).^2^ This economic loss threatened macroeconomic stability, food security, human capital development and private sector growth across the region.^3^

Various studies have assessed the systemic failures in the Ebola response, revealing that only about one third of countries in the world can prevent, detect and respond to public health emergencies. These studies have also shown, among other issues: (i) inadequate financing for pandemic preparedness; (ii) rigid instruments for emergency response; and (iii) slow and costly delivery of aid.^4^

To support countries to better understand their capacity gaps in pandemic preparedness and response, in 2016 the World Health Organization (WHO) established the joint external evaluation, a voluntary and collaborative process to assess a country’s capacity under the International Health Regulations (2005) to prevent, detect and rapidly respond to public health threats.^5^ However, this evaluation process does not fully address the financing for pandemic preparedness and response.

Even though the World Bank mobilized US$ 1.62 billion to support the countries hardest hit by the Ebola outbreak, these efforts were constrained by the lack of flexible instruments for channelling emergency resources in a timely fashion.^6^ To address this challenge, the World Bank Group is providing a variety of financing options to support pandemic preparedness and response.

The World Bank is supporting countries through corporate-level prioritization of pandemic preparedness and response. The International Development Association of the World Bank Group provides loans on soft terms and grants to the 77 poorest countries in the world. The association is replenished every three years.^7^ For the period that began in July 2017, the World Bank has committed to support at least 25 countries to develop multisectoral pandemic preparedness plans and to develop or strengthen multisectoral governance mechanisms to coordinate implementation of national pandemic preparedness plans.^8^

The World Bank is also engaging in strategic partnerships, for instance through its partnership with the governments of Australia and Japan, to support 13 countries across sub-Saharan Africa, Asia and the Caribbean to develop preparedness plans and coordination mechanisms.

The World Bank has also set up a Pandemic Emergency Financing Facility. In June 2017, the World Bank launched this facility to offer surge funding in the form of grants and insurance pay-outs, allowing funds to reach countries and international responders more quickly and effectively, and in so doing, prevent outbreaks from becoming pandemics.

The World Bank is also using high-level advocacy to strengthen pandemic preparedness and response. Since 2016, the World Bank – in partnership with the World Economic Forum and the Bill & Melinda Gates Foundation – has informed decision-makers at the national, regional and global levels about the importance and urgency of investing in pandemic preparedness. Two simulation exercises (October 2016 and January 2017) have informed pandemic preparedness discussions during the Annual Meeting of Ministers of Finance at the World Bank and chief executive officers in the private sector during the World Economic Forum Annual Meeting in Davos. 

The World Bank is also leveraging its convening role to mobilize resources for regional projects and initiatives, such as the Regional Disease Surveillance and Enhancement Project for West Africa and the World Bank’s collaboration with the Australian government to implement a regional technical assistance programme to ensure sustainability of health security in east Asia. In addition, the World Bank’s International Working Group on Financing Preparedness proposes ways in which national governments and development partners can collaborate to finance investments that will strengthen country preparedness and response capacities for health emergencies.

In February 2018, the World Bank in partnership with WHO, the Africa Centres for Disease Control and Prevention and the Bill & Melinda Gates Foundation will host a regional event to define the core elements of a multisectoral pandemic preparedness plan, including planning and coordination mechanisms. In addition, during the Prince Mahidol Award Conference^9^ to be held in January 2018 in Bangkok, the World Bank will host a series of meetings to share lessons learnt from country implementation, as well as models for financing pandemic preparedness.

To ensure the success of pandemic financing preparedness, countries should engage in dialogue on the importance of investing in such preparedness. The World Bank’s early successes need to continue through partnerships to renew and sustain national, regional and global commitment and investments for pandemic preparedness and response.
